# Primary Bilateral Macronodular Adrenal Hyperplasia Associated With *ARMC5* Variant and Pituitary Microadenoma

**DOI:** 10.1210/jcemcr/luaf288

**Published:** 2026-01-06

**Authors:** Lucía O’Connor-Ramiro, Pablo J Fernández, Julia Maroto, Ana Patiño-García, Javier Escalada, Carolina M Perdomo

**Affiliations:** Department of Endocrinology and Nutrition, Clínica Universidad de Navarra, Pamplona, Navarra 31008, Spain; Department of Endocrinology and Nutrition, Clínica Universidad de Navarra, Pamplona, Navarra 31008, Spain; Department of Biochemistry, Clínica Universidad de Navarra, Pamplona, Navarra 31008, Spain; Department of Pediatrics and Clinical Genetics Unit, Clínica Universidad de Navarra, Pamplona, Navarra 31008, Spain; IdiSNA (Instituto de Investigación en la Salud de Navarra), Pamplona, Navarra 31008, Spain; Department of Endocrinology and Nutrition, Clínica Universidad de Navarra, Pamplona, Navarra 31008, Spain; IdiSNA (Instituto de Investigación en la Salud de Navarra), Pamplona, Navarra 31008, Spain; Centro de Investigación Biomédica en Red-Fisiopatología de la Obesidad y Nutrición (CIBEROBN), Instituto de Salud Carlos III, Pamplona, Navarra 31008, Spain; Department of Endocrinology and Nutrition, Clínica Universidad de Navarra, Pamplona, Navarra 31008, Spain; IdiSNA (Instituto de Investigación en la Salud de Navarra), Pamplona, Navarra 31008, Spain; Centro de Investigación Biomédica en Red-Fisiopatología de la Obesidad y Nutrición (CIBEROBN), Instituto de Salud Carlos III, Pamplona, Navarra 31008, Spain

**Keywords:** *ARMC5* gene, primary bilateral macronodular adrenal hyperplasia, pituitary microadenoma

## Abstract

We report a case of primary bilateral macronodular adrenal hyperplasia (PBMAH) in a 63-year-old man with a novel germline armadillo repeat-containing protein 5 (*ARMC5)* variant of uncertain significance (c.2525T > C; p.Phe842Ser). Imaging and clinical findings revealed markedly enlarged bilateral adrenal glands and features of mild Cushing syndrome (CS). Clinical suspicion and recommendations from guidelines prompted genetic testing. Initial management focused on controlling comorbidities and monitoring hypercortisolism. Aberrant receptor testing was negative. Progression to overt CS prompted a nor-cholesterol scintigraphy scan, revealing higher uptake in the right adrenal gland. Right adrenalectomy was performed. Concurrent findings of hypogonadotropic hypogonadism and hyperprolactinemia led to the diagnosis of a pituitary microprolactinoma on magnetic resonance imaging. To our knowledge, this is the second reported case of PBMAH associated with a pituitary adenoma in the context of an *ARMC5* variant.

## Introduction

Primary bilateral macronodular adrenal hyperplasia (PBMAH) is characterized by multiple nonpigmented macronodules (>1 cm) [1]. Its morphological and clinical presentation is heterogeneous [2], and in some cases leads to cortisol overproduction and Cushing syndrome (CS). It accounts for less than 2% of endogenous CS and approximately 10% to 15% of adrenal CS cases [3]. Patients commonly present with mild autonomous cortisol secretion (MACS), which may progress to CS over time. PBMAH may be sporadic or caused by genetic mutations that promote abnormal adrenal growth [1], thereby affecting hormone regulation pathways. Germline armadillo repeat-containing protein 5 (*ARMC5*) mutations are a frequent genetic defect [1], responsible for most familial cases [3]. Their prevalence may reach 55% in overt CS and approximately 11% in mild CS [1].

## Case Presentation

A 63-year-old White man with a history of ulcerative colitis, depression, coxarthrosis, and previously well-controlled hypertension (off medication) was referred to our endocrinology clinic in September 2023 for a second opinion after a PBMAH diagnosis at another center. Before his first evaluation at our center, the patient had high 24-hour urinary cortisol levels (154.8 μg/24 hours [Système International (SI): 427 nmol/24 hours]) (reference range: <100 μg/24 hours [SI: <276 nmol/24 hours]; assessment performed by immunoassay), with suppressed adrenocorticotropic (ACTH) levels (<5 pg/mL [SI: 1.1 pmol/L]) (reference range, 10-60 pg/mL [SI: 2.2-13.2 pmol/L]). An abdominal magnetic resonance imaging (MRI) scan from December 2022 revealed bilateral adrenal enlargement (right: 45 × 43 × 60 mm; left: 50 × 30 × 62 mm).

## Diagnostic Assessment

In September 2023, on first evaluation at our center, the patient had a body mass index of 28.1, a waist circumference of 104 cm, and a blood pressure of 150/99 mm Hg; however, no typical signs of CS were evidenced. Family history was negative for adrenal disease. Laboratory analysis revealed endogenous hypercortisolism: late-night plasma cortisol of 11.45 µg/dL (SI: 316 nmol/L) (reference range, <5 µg/dL [SI: <138 nmol/L]; cortisol after 1-mg dexamethasone suppression test of 12 µg/dL (SI: 331 nmol/L) (reference range, <1.8 µg/dL [SI: <50 nmol/L] with suppressed ACTH. Furthermore, hypertension (nondipper pattern), osteoporosis, dyslipidemia, and prediabetes were diagnosed. In addition, suppressed renin levels were evidenced, with an aldosterone/renin ratio of 188. Metanephrines and catecholamines in 24-hour urine and in plasma were normal. Table 1 summarizes the hormonal assessment since the diagnosis of PBMAH.

**Table 1.** Patient's hormonal assessment over time

Evaluation date	Free urinary cortisol RR: <100 µg/24 h (SI: <276 nmol/24 h)	Basal cortisol RR: 6-18.4 µg/dL (SI: 166-508 nmol/L)	Basal prolactin RR: 4-15 ng/mL (SI: 4-15 µg/L)	Gonadotropins LH: RR 1.7-8.6 IU/L (SI: 1.7-8.6 IU/L) FSH: RR 1.5-12.4 IU/L (SI: 1.5-12.4 IU/L)	Free testosterone RR: 4.3-30.4 pg/mL (SI: 14.7-105.5 pg/mL)
December 2021	77.9 µg/24 h (215 nmol/24 h)	11.4 µg/dL (315 nmol/L)			
December 2022	154.8 µg/24h (427 nmol/24 h)	11.8 µg/dL (326 nmol/L)			
September 2023*^a^*	139.9 µg/24h (386 nmol/24 h)	15.6 µg/dL (430 nmol/L)	81.11 ng/mL (81.11 µg/L)	LH: 2.4 IU/L (2.4 IU/L) FSH: 9.3 IU/L (9.3 IU/L)	1.2 pg/mL (4.9 pg/mL)
October 2023			Cabergoline treatment		
February 2024	241.6 µg/24 h (667 nmol/24 h)	14.8 µg/dL (409 nmol/L)	4.57 ng/mL (4,57 µg/L)		5.1 pg/mL (18.7 pg/mL)
June 2024	444.8 µg/24 h (1228 nmol/24 h)	19.4 µg/dL (536 nmol/L)	2,7 ng/mL (2,7 µg/L)		
August 2024	Right adrenalectomy				
September 2024	245.5 µg/24 h (678 nmol/24 h)	5.2 µg/dL (143 nmol/L)	3.4 ng/mL (3.4 µg/L)		
January 2025	24.8 µg/24 h (68 nmol/24 h)	6.32 µg/dL (168 nmol/L)	3.3 ng/mL (3.4 µg/L)		
September 2025	61.7 µg/24 h (170 nmol/24 h)	10.68 µg/dL (294 nmol/L)	3.3 ng/mL (3.4 µg/L)		6 pg/mL (22.0 pg/mL)

Abbreviations: FSH, follicle-stimulating hormone; LH, luteinizing hormone; RR, reference range; SI, Système International.

^*a*^First assessment at our center.

**Figure 1.**
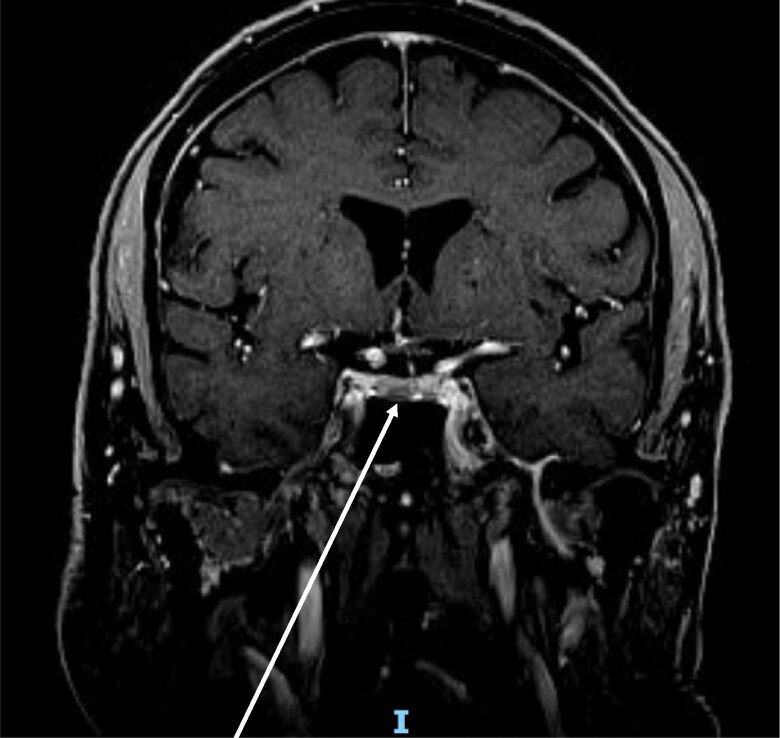
Gadolinium-enhanced pituitary magnetic resonance imaging (coronal view; T2).

Simultaneously, in the context of low libido, hypogonadotropic hypogonadism and hyperprolactinemia (plasma prolactin: 81.11 ng/mL [SI: 3540 pmol/L]) (reference range, 2-18 ng/mL [SI: 87-780 pmol/L]) were diagnosed. Other pituitary hormones were normal. Pituitary MRI revealed a T2-hypointense pituitary microadenoma (8 × 4 mm) (Fig. 1).

Cardiovascular risk factors were controlled with medication. Osteoporosis was attributed both to hypogonadism and hypercortisolism; thus, treatment was recommended. Given mild hypercortisolism, we tested for aberrant adrenal receptors, limiting stimulation tests to those with available targeted treatment; no aberrant receptors were detected (Table 2). During follow-up in October 2023, a computed tomography scan revealed an increase in adrenal size (right: 43 × 42 × 83 mm; left: 52 × 32 × 32 × mm). After consultation with our genetics department, a next-generation sequencing panel analyzing 9 genes (aryl hydrocarbon receptor interacting protein [*AIP*], adenomatous polyposis coli [*APC*], *ARMC5*, cyclin dependent kinase inhibitor 1B [*CDKN1B*], fumarate hydratase [*FH*], guanine nucleotide binding protein α stimulating [*GNAS*], melanocortin 2 receptor [*MC2R*], multiple endocrine neoplasia 1 [*MEN1*], and protein kinase cyclic adenosine monophosphate–activated catalytic subunit α [*PRKACA*] was requested. A heterozygous *ARMC5* variant (c.2525T > C; p.Phe842Ser) was identified. Genetic counseling was offered.

**Table 2.** Aberrant receptor testing

Date and stimuli	Nov. 15, 2023 8:43 h Standing position	Nov. 15, 2023 11:30 h Standard meal test	Nov. 16, 2023 8:50 h TRH 200 mcg i.v.	Nov. 17, 2023 8:46 h GnRH 100 mcg i.v.
Basal cortisol	12.2 µg/dL (337 nmol/L)	12.12 µg/dL (334 nmol/L)	13.47 µg/dL (372 nmol/L)	11.95 µg/dL (330 nmol/L)
Cortisol at 30 min	13.14 µg/dL (363 nmol/L)	12.16 µg/dL (336 nmol/L)	12.96 µg/dL (358 nmol/L)	11.96 µg/dL (330 nmol/L)
Cortisol at 60 min	14 µg/dL (387 nmol/L)	11.43 µg/dL (316 nmol/L)	13.05 µg/dL (360 nmol/L)	11.91 µg/dL (329 nmol/L)
Cortisol at 90 min	14.55 µg/dL (402 nmol/L)	10.94 µg/dL (302 nmol/L)	13.07 µg/dL (361 nmol/L)	11.74 µg/dL (324 nmol/L)
Cortisol at 120 min	14.97 µg/dL (413 nmol/L)	10.75 µg/dL (297 nmol/L)	12.57 µg/dL (347 nmol/L)	11.89 µg/dL (328 nmol/L)

Abbreviations: GnRH, gonadotropin-releasing hormone; i.v., intravenously; TRH, thyrotropin-releasing hormone.

## Treatment

**Figure 2.**
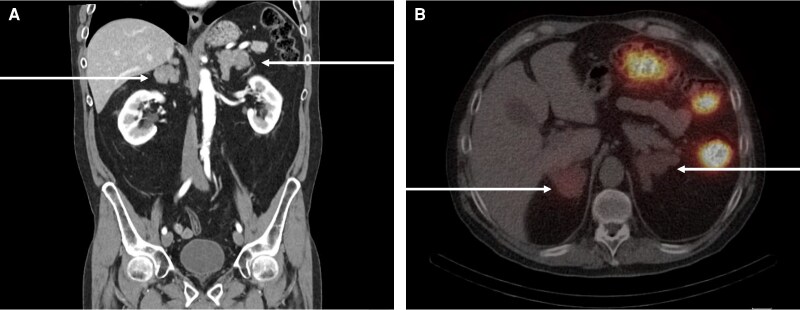
A, Computed tomography of the abdomen showing the massively enlarged bilateral adrenal glands. B, Nor-cholesterol scintigraphy revealed a greater uptake in the right adrenal gland.

Pharmacological treatment for comorbidities initially included the following: spironolactone 100 mg daily (suspected primary hyperaldosteronism), pravastatin 20 mg daily, alendronic acid 70 mg weekly, vitamin D3 2000 IU (6 drops at breakfast), and cabergoline 0.5 mg (half a tablet, twice weekly). In December 2023, after the captopril challenge test, primary hyperaldosteronism was ruled out, and spironolactone was modified to losartan. In June 2024, free urinary cortisol levels exceeded 3 times the upper limit, and a nor-cholesterol scintigraphy was performed to guide unilateral adrenalectomy. The scan showed greater uptake in the right adrenal gland (Fig. 2B). Right adrenalectomy was performed in August 2024 without complications. Postoperatively, the patient developed adrenal insufficiency; basal cortisol was 5.2 µg/dL (143 nmol/L) (reference range, >10 µg/dL [SI: >275 nmol/L] with a cortisol response to ACTH stimulation of 17 µg/dL (reference range, >18 µg/dL [SI: >496 nmol/L]), thus oral hydrocortisone was indicated.

## Outcome and Follow-up

The patient received glucocorticoid support until March 2025, after demonstrating a basal cortisol level of 10.68 µg/dL. At his most recent medical consultation, he reported improved mood, decreased fatigue, and increased libido. He reports having enough energy to engage in at least 30 minutes of daily exercise. Hypertension treatment was suspended because ambulatory blood pressure levels were normal. A second pituitary MRI was performed in September 2025, and the disappearance of the adenoma was evidenced.

## Discussion

We report a case of PBMAH with overt CS in a patient carrying a previously undescribed germline *ARMC5* variant (c.2525T > C; p.Phe842Ser), classified as of uncertain significance. PBMAH is a rare adrenal disorder, though its true prevalence may be underestimated [3]. Adrenal incidentalomas occur in 1% to 5% of the population, with 2.7% to 10% being bilateral [4]. MACS is present in 35% to 40% of these bilateral incidentalomas, and some may represent PBMAH. Table 3 summarizes the key features of PBMAH.

**Table 3.** Key features of primary bilateral macronodular adrenal hyperplasia

Aspect	Key features	References
Disease definition	Bilateral enlargement of adrenal cortex with multiple macronodules producing cortisol autonomously Heterogeneous clinical presentation (subclinical or overt hypercortisolism)	[1-4, 6, 20]
Epidemiology	Rare cause of adrenal CS; usually in middle-aged adults	[1-4, 6, 20]
Etiology	Multifactorial: aberrant expression of G protein–coupled and other hormone receptors in adrenal nodules causing cAMP/PKA activation (eg, vasopressin, LH/hCG, GIP, β-adrenergic, angiotensin); focal/paracrine intra-adrenal production of ACTH by steroidogenic cells; somatic and germline genetic alterations (notably ARMC5) acting as drivers in a subset.	[1-3, 7-9]
Genetic basis	Predominantly sporadic; familial forms also reported *ARMC5* most common (located at 16p11.2); other rare genes: *KDM1A*, *GNAS*, *MEN1*, *FH*, *APC*, *PRKACA*, *PDE11A*	[5-9, 13, 15, 20]
ARMC5 phenotype	Larger adrenal glands; more frequent CS; occasional association with extra-adrenal tumors (eg, meningiomas)	[5-9, 11-13, 20]
Genotyping indication	Bilateral adrenal enlargement with CS/MACS; family history; first-degree family screening	[7-10, 20]
Management	Unilateral adrenalectomy preferred; bilateral only if refractory. Steroidogenesis inhibitors or glucocorticoid receptor antagonists may be used. Aberrant receptor blockade occasionally effective. Lifelong follow-up required	[1, 2, 16, 18, 20]
Prognosis	Progressive disease; morbidity from cortisol excess; improved outcomes with early detection and surgery	[1, 2, 4, 6, 20]

Abbreviations: ACTH, adrenocorticotropin; *APC*, adenomatous polyposis coli; *ARMC5*, armadillo repeat containing 5; cAMP, cyclic adenosine monophosphate; CS, Cushing syndrome; *FH*, fumarate hydratase; *GNAS*, guanine nucleotide binding protein, α stimulating; *KDM1A*, lysine demethylase 1A; *MEN1*, multiple endocrine neoplasia 1; MACS, mild autonomous cortisol secretion; PBMAH, primary bilateral macronodular adrenal hyperplasia; *PDE11A*, phosphodiesterase 11A; PKA, protein kinase A; *PRKACA*, protein kinase cAMP-activated catalytic subunit α.

While most PBMAH cases are sporadic, a substantial proportion involve germline *ARMC5* mutations [5]. More than 265 mutations have been identified [4, 6], including several familial cases with autosomal dominant inheritance [1, 2]. *ARMC5* is the primary genetic cause of PBMAH, accounting for up to 80% of familial and 30% of sporadic cases [1-3]. In an Italian cohort, 18.8% of patients with MACS carried pathogenic *ARMC5* variants [7].

The c.2525T > C; p.(Phe842Ser) *ARMC5* variant was initially classified as a variant of unknown clinical significance according to the American College of Medical Genetics and Genomics [8] on fulfilment of the PM2 (“absent from controls (or at extremely low frequency if recessive) in Exome Sequencing Project, 1000 Genomes Project, or Exome Aggregation Consortium”) and PP4 (“patient's phenotype or family history is highly specific for a disease with a single genetic etiology”). To definitively relate the presence of this variant with disease, segregation analysis of family members will be performed for fulfillment of PS2 and/or PP1 criteria, which would lead to final classification of the variant as “likely pathogenic.”

Of particular interest in this case is the co-occurrence of a prolactin-secreting pituitary microadenoma. This is the second case report of a patient with both PBMAH and a pituitary adenoma, presenting with a germline *ARMC5* variant [9]. *ARMC5* is believed to act as a tumor suppressor gene, inhibiting abnormal cell proliferation [5]. It encodes a protein predominantly located in the cytoplasm, whose function depends on interactions with other proteins [5]. Loss of *ARMC5* function may contribute to tumor formation through a 2-hit mechanism—a germline mutation followed by a somatic event that affects the second allele.

Inactivation of *ARMC5* in the adrenal cortex is associated with progressive dedifferentiation of adrenocortical cells, growth of bilateral masses, and inefficient steroidogenesis [6]. Beyond PBMAH, *ARMC5* mutations have been associated with other neoplasms, including intracranial meningiomas [1, 2]. Its high expression in the pituitary gland [10] suggests a potential, though unconfirmed, role in pituitary tumorigenesis. It remains unknown whether this specific variant predisposes to microadenoma development. Other endocrine comorbidities, such as primary hyperaldosteronism, hyperparathyroidism, and multinodular goiter, have also been described in carriers of *ARMC5* pathogenic variants [11]. Likewise, associations with breast, thyroid, and parathyroid cancer have been reported [11, 12], although more studies are needed to clarify these links.

Other genetic causes of PBMAH include inactivation of lysine demethylase type 1A (*KDM1A*), which has been linked to hereditary food-dependent CS (glucose-dependent insulinotropic peptide–dependent PBMAH) [13]. This mutation is also associated with adrenal myelolipoma, monoclonal gammopathy of undetermined significance, and multiple myeloma. PBMAH may also result from alterations in *MC2R*, *PRKACA*, and phosphodiesterase (*PDE)11A* genes [1, 2], and it can occur as part of familial tumor syndromes such as McCune-Albright syndrome (*GNAS*), MEN1, familial *APC*, or hereditary leiomyomatosis and renal cell carcinoma (*FH* gene variant). Genetic testing may aid in the earlier diagnosis and management of PBMAH [5], though there are currently no established criteria for screening. Given the delayed onset and frequent lack of symptoms, genetic screening of first-degree relatives may help identify individuals at risk [1].

Assessment of comorbidities associated with cortisol excess, such as diabetes, hypertension, and osteoporosis, is also crucial. Aberrant adrenal receptors are found in approximately 30% of cases of bilateral and unilateral adrenal hyperplasia [2] and can be identified through stimulation tests that evaluate abnormal cortisol responses [1]. Although receptor blockade rarely achieves long-term control, it may offer other treatment options [2, 14]. Testing under dexamethasone suppression may help minimize ACTH interference [1].

In patients with clinically significant hypercortisolism, surgical intervention is often necessary [1, 2]. Bilateral adrenalectomy offers definitive control but requires lifelong steroid replacement and carries substantial risk [2]. Therefore, unilateral adrenalectomy is preferred when possible, targeting the gland with a higher uptake on iodocholesterol scintigraphy of the larger gland. In our case, the glands were similar in size, and scintigraphy guided the decision. Adrenal venous sampling (AVS) is generally unhelpful in asymmetric PBMAH since the larger gland usually secretes more cortisol in AVS [15], though it may be helpful in symmetric presentations [14].

Medical therapy, such as ketoconazole or mitotane, can be used before surgery or for long-term management in patients who are not surgical candidates [1, 2]. Low-dose ketoconazole has been successfully used for up to 10 years in selected cases [16]. Receptor-targeted treatments, such as propranolol, octreotide, long-acting gonadotropin–releasing hormone (GnRH) agonists, or angiotensin receptor blockers, may be considered when appropriate [2]. However, their effectiveness varies, and they may not result in clinical improvement. Mifepristone (a glucocorticoid receptor antagonist) and osilodrostat (an adrenostatic agent) may also be considered medical options for PBMAH when surgery is not feasible [17, 18].

Lifelong follow-up is essential [19, 20], as recurrence of hypercortisolism is very likely; it occurs in 10% to 68% of cases after unilateral adrenalectomy, sometimes years later [2, 18]. In selected cases, total adrenalectomy of the larger gland, combined with partial resection of the contralateral gland, may reduce the risk of recurrence. Our patient will be evaluated every 6 to 12 months. The hypothalamic-pituitary-adrenal axis and hypercortisolism will be assessed periodically. The withdrawal of cabergoline is planned; thus, MRI revealed the disappearance of the prolactinoma after 18 months of treatment. In conclusion, this case contributes to the expanding phenotype of *ARMC5*-associated disease and raises questions about its role in the pathogenesis of pituitary tumors. Although causality cannot be confirmed, this association warrants further investigation.

## Learning Points

- PBMAH is a complex endocrine disorder with variable clinical presentations frequently associated with *ARMC5* mutations.
- *ARMC5* pathogenic variants are associated with a more severe phenotype of PBMAH.
- We have described a variant of uncertain significance of the *ARMC5* gene in a patient with PBMAH and pituitary abnormalities. It is unknown if this variant is associated with these comorbidities.
- The spectrum of *ARMC5*-related tumors must be outlined; in the meantime, patients should be monitored for other neoplastic entities.
- *ARMC5* screening might be recommended in patients with PBMAH and MACS or CS.

## Contributors

All authors made individual contributions to the authorship. L.O., P.F., and C.P. were involved in the case presentation, diagnosis, and patient care, as well as the creation of graphs. J.M., A.P., and J.E. were involved in case diagnosis. J.E. was involved in case follow-up. All authors reviewed and approved the final draft.

## Data Availability

Some or all datasets generated during and/or analyzed during the current study are not publicly available but are available from the corresponding author on reasonable request.

## Abbreviations

ACTH: adrenocorticotropin
*APC*: adenomatous polyposis coli
*ARMC5*: armadillo repeat containing 5
AVS: adrenal venous sampling
cAMP: cyclic adenosine monophosphate
CS: Cushing syndrome
*FH*: fumarate hydratase
FSH: follicle-stimulating hormone
*GNAS*: guanine nucleotide binding protein, α stimulating
GnRH: gonadotropin–releasing hormone
*KDM1A*: lysine demethylase 1A
LH: luteinizing hormone
*MEN1*: multiple endocrine neoplasia 1
MACS: mild autonomous cortisol secretion
MRI: magnetic resonance imaging
PBMAH: primary bilateral macronodular adrenal hyperplasia
*PDE11A*: phosphodiesterase 11A
PKA: protein kinase A
*PRKACA*: protein kinase cAMP-activated catalytic subunit α
